# Genetic invalidation of Lp-PLA_2_ as a therapeutic target: Large-scale study of five functional Lp-PLA_2_-lowering alleles

**DOI:** 10.1177/2047487316682186

**Published:** 2016-12-08

**Authors:** John M Gregson, Daniel F Freitag, Praveen Surendran, Nathan O Stitziel, Rajiv Chowdhury, Stephen Burgess, Stephen Kaptoge, Pei Gao, James R Staley, Peter Willeit, Sune F Nielsen, Muriel Caslake, Stella Trompet, Linda M Polfus, Kari Kuulasmaa, Jukka Kontto, Markus Perola, Stefan Blankenberg, Giovanni Veronesi, Francesco Gianfagna, Satu Männistö, Akinori Kimura, Honghuang Lin, Dermot F Reilly, Mathias Gorski, Vladan Mijatovic, on behalf of the CKDGen consortium, Patricia B Munroe, Georg B Ehret, on behalf of the International Consortium for Blood Pressure, Alex Thompson, Maria Uria-Nickelsen, Anders Malarstig, Abbas Dehghan, on behalf of the CHARGE inflammation working group, Thomas F Vogt, Taishi Sasaoka, Fumihiko Takeuchi, Norihiro Kato, Yoshiji Yamada, Frank Kee, Martina Müller-Nurasyid, Jean Ferrières, Dominique Arveiler, Philippe Amouyel, Veikko Salomaa, Eric Boerwinkle, Simon G Thompson, Ian Ford, J Wouter Jukema, Naveed Sattar, Chris J Packard, Abdulla al Shafi Majumder, Dewan S Alam, Panos Deloukas, Heribert Schunkert, Nilesh J Samani, Sekar Kathiresan, on behalf of the MICAD Exome consortium, Børge G Nordestgaard, Danish Saleheen, Joanna MM Howson, Emanuele Di Angelantonio, Adam S Butterworth, John Danesh

**Affiliations:** 1 MRC/BHF Cardiovascular Epidemiology Unit, Department of Public Health and Primary Care, University of Cambridge, UK; 2 Departments of Medicine and Genetics, Washington University School of Medicine, St Louis, USA; 3 Department of Public Health and Primary Care, University of Cambridge, UK; 4 Department of Neurology, Innsbruck Medical University, Austria; 5 Copenhagen University Hospital, University of Copenhagen, Denmark; 6 University of Glasgow, UK; 7 Leiden University Medical Centre, Netherlands; 8 University of Texas Health Science Center Houston, USA; 9 THL-National Institute for Health and Welfare, Helsinki, Finland; 10 Institute of Molecular Medicine FIMM, University of Helsinki, Finland; 11 Department of Health, National Institute for Health and Welfare, Helsinki, Finland; 12 Department of General and Interventional Cardiology, University Heart Centre Hamburg, Germany; 13 University Medical Centre Hamburg Eppendorf, Hamburg, Germany; 14 Research Centre, Department of Clinical and Experimental Medicine, University of Insubria, Varese, Italy; 15 Department of Epidemiology and Prevention, IRCCS Istituto Neurologico Mediterraneo Neuromed, Pozzilli, Italy; 16 Department of Molecular Pathogenesis, Medical Research Institute, Tokyo Medical and Dental University (TMDU), Japan; 17 Section of Computational Biomedicine, Department of Medicine, Boston University School of Medicine, USA; 18 The NHLBI’s Framingham Heart Study, Framingham, USA; 19 Merck Research Laboratories, Genetics and Pharmacogenomics, Boston, USA; 20 Department of Genetic Epidemiology, University of Regensburg, Germany; 21 Department of Nephrology, University Hospital Regensburg, Germany; 22 Department of Life and Reproduction Sciences, University of Verona, Italy; 23 Clinical Pharmacology and The Genome Centre, William Harvey Research Institute, Barts and The London School of Medicine and Dentistry, Queen Mary University of London, UK; 24 NIHR Barts Cardiovascular Biomedical Research Unit, Queen Mary University of London, UK; 25 Center for Complex Disease Genomics, McKusick-Nathans Institute of Genetic Medicine, Johns Hopkins University School of Medicine, Baltimore, USA; 26 Cardiology, Department of Medicine, Geneva University Hospital, Switzerland; 27 Institute of Social and Preventive Medicine (IUMSP), Centre Hospitalier Universitaire Vaudois and University of Lausanne, Lausanne, Switzerland; 28 UCB, Brussels, Belgium; 29 Clinical Research, Pfizer Worldwide R&D, Cambridge, USA; 30 Clinical Research, Pfizer Worldwide R&D, Sollentuna, Sweden; 31 Department of Epidemiology, Erasmus University Medical Centre, Rotterdam, The Netherlands; 32 Merck Research Laboratories, Cardiometabolic Disease, Kenilworth, USA; 33 CHDI Management/CHDI Foundation, Princeton, USA; 34 Department of Gene Diagnostics and Therapeutics, Research Institute, National Centre for Global Health and Medicine, Tokyo, Japan; 35 Department of Human Functional Genomics, Life Science Research Centre, Mie University, Japan; 36 UKCRC Centre of Excellence for Public Health, Queens University, Belfast, Ireland; 37 Institute of Genetic Epidemiology, Helmholtz Zentrum München - German Research Centre for Environmental Health, Neuherberg, Germany; 38 Institute of Medical Informatics, Biometry and Epidemiology, Ludwig-Maximilians-Universität, Munich, Germany; 39 DZHK (German Centre for Cardiovascular Research), partner site Munich Heart Alliance, Munich, Germany; 40 Department of Medicine I, Ludwig-Maximilians-University Munich, Germany; 41 Department of Epidemiology, UMR 1027-INSERM, Toulouse University-CHU Toulouse, France; 42 Department of Epidemiology and Public Health, EA 3430, University of Strasbourg and Strasbourg University Hospital, France; 43 Department of Epidemiology and Public Health, Institut Pasteur de Lille, France; 44 Human Genetics Center, University of Texas Health Science Center at Houston, USA; 45 National Institute of Cardiovascular Diseases, Sher-e-Bangla Nagar, Dhaka, Bangladesh; 46 Centre for Global Health Research, St Michael Hospital, Toronto, Canada; 47 William Harvey Research Institute, Barts and The London School of Medicine and Dentistry, Queen Mary University of London, UK; 48 Deutsches Herzzentrum München, Technische Universität München, Germany; 49 Department of Cardiovascular Sciences, University of Leicester and National Institute for Health Research Leicester Cardiovascular Biomedical Research Unit, UK; 50 Broad Institute, Cambridge and Massachusetts General Hospital, Boston, USA; 51 University of Pennsylvania, Philadelphia, USA; 52 Wellcome Trust Sanger Institute, Hinxton, Cambridge, UK; 53 British Heart Foundation Cambridge Centre of Excellence, University of Cambridge, Cambridge, UK; 54 National Institute of Health Research Blood and Transplant Research Unit in Donor Health and Genomics, University of Cambridge, Cambridge, UK

**Keywords:** Human genetics, target validation, coronary heart disease, lipoprotein-associated phospholipase A_2_, darapladib

## Abstract

**Aims:**

Darapladib, a potent inhibitor of lipoprotein-associated phospholipase A_2_ (Lp-PLA_2_), has not reduced risk of cardiovascular disease outcomes in recent randomized trials. We aimed to test whether Lp-PLA_2_ enzyme activity is causally relevant to coronary heart disease.

**Methods:**

In 72,657 patients with coronary heart disease and 110,218 controls in 23 epidemiological studies, we genotyped five functional variants: four rare loss-of-function mutations (c.109+2T > C (rs142974898), Arg82His (rs144983904), Val279Phe (rs76863441), Gln287Ter (rs140020965)) and one common modest-impact variant (Val379Ala (rs1051931)) in *PLA2G7,* the gene encoding Lp-PLA_2_. We supplemented de-novo genotyping with information on a further 45,823 coronary heart disease patients and 88,680 controls in publicly available databases and other previous studies. We conducted a systematic review of randomized trials to compare effects of darapladib treatment on soluble Lp-PLA_2_ activity, conventional cardiovascular risk factors, and coronary heart disease risk with corresponding effects of Lp-PLA_2_-lowering alleles.

**Results:**

Lp-PLA_2_ activity was decreased by 64% (*p* = 2.4 × 10^–25^) with carriage of any of the four loss-of-function variants, by 45% (*p* < 10^–300^) for every allele inherited at Val279Phe, and by 2.7% (*p* = 1.9 × 10^–12^) for every allele inherited at Val379Ala. Darapladib 160 mg once-daily reduced Lp-PLA_2_ activity by 65% (*p* < 10^–300^). Causal risk ratios for coronary heart disease per 65% lower Lp-PLA_2_ activity were: 0.95 (0.88–1.03) with Val279Phe; 0.92 (0.74–1.16) with carriage of any loss-of-function variant; 1.01 (0.68–1.51) with Val379Ala; and 0.95 (0.89–1.02) with darapladib treatment.

**Conclusions:**

In a large-scale human genetic study, none of a series of Lp-PLA_2_-lowering alleles was related to coronary heart disease risk, suggesting that Lp-PLA_2_ is unlikely to be a causal risk factor.

## Introduction

Lipoprotein-associated phospholipase A_2_ (Lp-PLA_2_), an enzyme expressed by inflammatory cells in atherosclerotic plaques, is carried in the circulation bound predominantly to low-density lipoprotein (LDL).^1,2^ Lp-PLA_2_ (also called platelet-activating factor acetyl hydrolase) hydrolyses oxidized phospholipids to yield pro-inflammatory products implicated in endothelial dysfunction, plaque inflammation and formation of necrotic core in plaque.^1^ Observational^3^ and experimental studies in humans and animals have suggested that Lp-PLA_2_ could be a valid therapeutic target, postulating this enzyme to link oxidative modification of LDL and development of inflammatory responses to arterial intima.^1^ Previous studies have investigated genetic variants altering Lp-PLA_2_ function in relation to coronary heart disease (CHD) risk.^4,5^ However, these studies have generally yielded inconclusive, or conflicting results,^4,5^ perhaps due to limited statistical power and due to limited knowledge about variants altering Lp-PLA_2_ function (e.g. previous studies have been able to consider only one loss-of-function variant in *PLA2G7*, the gene encoding Lp-PLA_2_).

However, two phase 3 randomized trials of darapladib, a potent inhibitor of Lp-PLA_2_ activity, have not shown reductions in cardiovascular risk.^6,7^ These results could, at least in part, have been due to features of the trials. One of the phase 3 trials was restricted to patients recently hospitalized with acute coronary syndromes,^6^ yet many cardiovascular events occurring early after acute coronary syndromes may relate to thrombotic mechanisms and not be modifiable through Lp-PLA_2_ inhibition. Trials used statins as background therapy, so any Lp-PLA_2_ inhibition achieved with statins could have reduced any incremental benefits of darapladib. Trials could not assess the effects of prolonged Lp-PLA_2_ inhibition because they recorded only about 3–4 years of median follow-up.^6,7^

An alternative explanation is that darapladib did not reduce cardiovascular risk because Lp-PLA_2_ is not a causal risk factor in cardiovascular disease. We tested this possibility by investigating natural loss of Lp-PLA_2_ activity. Studies of Lp-PLA_2_-lowering alleles should complement randomized trials of darapladib because genotypes are fixed at conception, avoiding potential distorting effects of pre-existing disease and medication usage. Furthermore, Lp-PLA_2_-lowering alleles should produce lifelong, rather than shorter-term, Lp-PLA_2_ inhibition.

In over 260,000 participants of European, South Asian, or East Asian ancestries, we studied five functional variants in *PLA2G7*. We compared effects of Lp-PLA_2_-lowering alleles on soluble Lp-PLA_2_ activity, conventional cardiovascular risk factors and CHD risk with corresponding effects of darapladib, using results from randomized trials.

## Methods

### Study design

**Figure 1.**
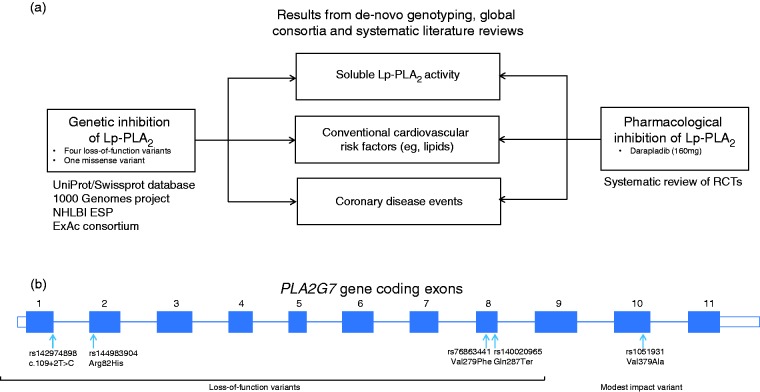
Summary of study design. (a) Flow chart of study design. (b) Exonic structure of the *PLA2G7* gene and location of variants used in this study. ExAc: Exome Aggregation consortium; NHLBI ESP: National Heart Lung and Blood Institute Exome Sequencing Project; Lp-PLA_2_: lipoprotein-associated phospholipase A_2_; RCT: randomized controlled trial; UniProt/Swissprot: manually annotated and reviewed section of the Universal Protein resource database.

Figure 1 summarizes the study approach. Table 1 provides definitions and sources of data used. First, we identified four loss-of-function mutations and one missense variant in *PLA2G7* suggested by previous experimental and bioinformatics studies, thereby developing an allelic series for Lp-PLA_2_ activity. Second, we assessed associations of these variants – both singly and in combination – with soluble Lp-PLA_2_ activity, conventional cardiovascular risk factors and CHD risk in people of European, South Asian or East Asian ancestries. Third, we compared associations of Lp-PLA_2_-lowering alleles with the aforementioned traits and CHD risk with the effects of darapladib treatment through a systematic review of randomized trials.

**Table 1.** Definitions and source of contributing data for the main study outcome.

**Lp-PLA_2_ assessment tool**	**Val279Phe, loss-of-function variant common in East Asians**	**Four loss-of-function variants^a^, rare in Europeans and South Asians**		**Val379Ala, modest impact variant**				**Darapladib**
Data sources	Systematic review: up to 12 East Asian studies	De-novo genotyping and participant-level data: up to eight European or South Asian ancestry studies from the CHD Exome^+^ consortium^19-26^ De-novo genotyping and study-level data: up to 15 European ancestry studies from the MICAD Exome consortium^38^ and three European ancestry studies from the CHARGE Consortium^17,29^ Plus publicly available consortium data						Systematic review: up to five randomized clinical trials^6,7,39-41^ from a systematic review
**Endpoint**	**Number of studies and unique individuals contributing to analyses; *n* total *or* cases/controls**							
**Coronary heart disease** ^b^	Seven East Asian studies**^c^**	Eight European or South Asian ancestry studies from the CHD Exome^+^ consortium^19-26^ ^d^	15 European ancestry studies from the MICAD Exome consortium^38^ ^c^	Eight European or South Asian ancestry studies from the CHD Exome^+^ consortium^19-26^ ^d^	Eight European ancestry studies from the MICAD Exome consortium^38^ ^c^	14 European ancestry studies from the CARDIoGRAM consortium^27^ ^e^	Four European or South Asian ancestry studies from the C4D consortium^28^ ^e^	Two phase 3 randomized clinical trials of darapladib^6,7^ ^c^
	10,088 cases 15,199 controls	35,829 cases 44,948 controls	35,533 cases 64,130 controls	32,196 cases 41,464 controls	14,976 cases 32,084 controls	20,315 cases 58,419 controls	15,420 cases 15,062 controls	3364 cases 25,490 non-cases
**Lp-PLA_2_ activity** ^f^	12 East Asian studies^c^	One European ancestry study from the CHD Exome^+^ consortium^25^ ^d^	One European ancestry study from the CHARGE consortium^29^ ^c^	Two European ancestry studies from the CHD Exome^+^ consortium^22,23,25^ ^d^		Three European ancestry studies from the CHARGE consortium^17^ ^c^		Three phase 2 randomized clinical trials^39-41^ ^c^
	8468	1240	8564	2173		11,662		854

### Genetic variants

We defined loss-of-function variants as non-synonymous variants with in vitro or in vivo evidence demonstrating complete lack of Lp-PLA_2_ activity or sequence changes expected to abolish Lp-PLA_2_ function (e.g. nonsense variants or mutations in essential splice sites). We selected variants through a systematic search for loss-of-function variants using the UniProt database,^8^ the Exome Aggregation Consortium database (Cambridge, MA, USA; URL: http://exac.broadinstitute.org (accessed November 2014)),^9^ studies of site-directed mutagenesis^10-12^ and results from targeted gene sequencing.^13^ Among the full set of variants identified (Supplementary Material online Table 1), we selected the following variants that could be detected in the 1000 Genomes^14^ or the NHLBI Exome Sequencing Project^15^ projects (and, hence, potentially studied at the population level): the splice site mutation 109+2T>C (rs142974898); two non-synonymous variants – Arg82His (rs144983904) and Val279Phe (rs76863441); and the nonsense variant Gln287Ter (rs140020965). These loss-of-function variants are rare in European and South Asian ancestry populations, whereas carriage of 279Phe is common in East Asian ancestry populations and abolition of Lp-PLA_2_ activity is well documented.^16^ Additionally, we studied Val379Ala (rs1051931), a functional variant common in European ancestry populations, which lowers Lp-PLA_2_ activity only modestly,^10,17^ in contrast with the much stronger Lp-PLA_2_-lowering achieved by the loss-of-function variants described above.

### Samples and data for genetic studies

We aimed to maximize study power and comprehensiveness by using the following complementary approaches to generate new data on, as well as to collate systematically existing relevant information about, the *PLA2G7* variants mentioned above: (1) we conducted de-novo genotyping for 72,657 CHD patients and 110,218 controls (the majority of whom also had information available on some cardiovascular risk factors); (2) we accessed non-overlapping summary-level data from the only known global genetics consortium of CHD,^18^ yielding information on a further 35,735 CHD patients and 73,481 controls; (3) we conducted a systematic review (supplemented by provision of tabular data from each study investigator) of published East Asian CHD studies of Val279Phe because these studies were not represented in the global CHD consortium, yielding information on a further 10,088 CHD cases and 15,199 controls; (4) we accessed summary-level data from the largest available global genetics consortium on each of several relevant cardiovascular risk factors (e.g. Lp-PLA_2_ activity, conventional lipids, blood pressure), yielding information on 489,045 participants. Each of these sources of information is summarized below and in Table 1, with a key in Table 1’s legend denoting the level of data detail available for each source (e.g. individual-participant data versus tabular study-level results).

#### Coronary heart disease outcomes

For CHD outcomes, we had access to data for a total of 92,995 patients and 162,228 controls. For 182,875 of these participants (72,657 CHD patients, 110,218 controls), we did de-novo genotyping of the four loss-of-function variants (c.109 + 2T > C, Arg82His, Val279Phe, Gln287Ter) and Val379Ala using customized Exome arrays (Illumina, CA, USA) by technicians masked to the phenotypic status of the participants’ samples. For 35,829 CHD cases, 44,948 controls in eight studies, we had access to individual-participant data. The eight studies were: the Bangladesh Risk of Acute Vascular Events Study,^19^ Copenhagen City Heart Study,^20^ Copenhagen Ischemic Heart Disease/Copenhagen General Population Study,^20^ European Prospective Investigation into Cancer and Nutrition-Cardiovascular Disease Study (EPIC-CVD),^21^ MONICA Risk, Genetics, Archiving, and Monograph (MORGAM) study,^22,23^ Pakistan Risk of Myocardial Infarction Study,^24^ Pravastatin in elderly individuals at risk of vascular disease (PROSPER) trial^25^ and the West of Scotland Coronary Prevention Study^26^ (these eight studies are collectively called the ‘CHD Exome^+^ consortium’). For 15 additional studies (collectively called the ‘MICAD consortium’) we used similar genotyping methods to those described above but did not genotype c.109+2T>C and had access only to study-level data. We supplemented de-novo data on Val379Ala with non-overlapping consortium-level results from a further 35,735 CHD patients and 73,481 controls in the transatlantic Coronary Artery Disease Genome-wide Replication and Meta-analysis^27^ and Coronary Artery Disease Genetics^28^ consortia (Table 1). We obtained tabular data on Val279Phe from seven East Asian studies involving a total of 10,088 CHD cases and 15,199 controls, identified through systematic review (text and Table 5 in Supplementary Material online). About 90% of CHD patients in our genetic analysis had myocardial infarction or other major acute coronary events; the remainder had angiographic evidence alone (e.g. >50% coronary stenosis; Supplementary Tables 2 and 5).

#### Lp-PLA_2_ activity

For 13,835 participants, we had information on functional variants in *PLA2G7* and Lp-PLA_2_ activity, using data from de-novo genotyping in MORGAM^22,23^ and PROSPER,^25^ supplemented by published data from the CHARGE Consortium (i.e. from the Atherosclerosis Risk in Communities study,^29^ Cardiovascular Health Study,^17^ Framingham Heart Study^17^ and Rotterdam study,^17^ and from 12 East Asian studies identified through the systematic review described above (Table 1; Supplementary Material text, Figure 1 and Tables 2 and 3).

#### Conventional cardiovascular risk factors

For 177,343 participants, we had information on functional variants in *PLA2G7* and conventional cardiovascular risk factors and several other traits, including circulating concentrations of LDL-cholesterol, high-density lipoprotein (HDL)-cholesterol, triglycerides, glucose, insulin and C-reactive protein, and values of systolic and diastolic blood pressure, body-mass index and estimated glomerular filtration rate. Again, we supplemented data from our de-novo genotyping, with information from existing global genetics consortia (Table 1; Supplementary Tables 2 to 4).

### Randomized trials of darapladib

To compare genetic associations with effects of pharmacological Lp-PLA_2_ inhibition, we conducted a systematic review to identify randomized placebo-controlled trials of darapladib that had reported on Lp-PLA_2_ activity, conventional risk factors and/or CHD events (Supplementary Material). CHD events in the trials were defined as fatal CHD, myocardial infarction or urgent revascularization, as recorded in STABILITY (Stabilization of Atherosclerotic Plaque by Initiation of Darapladib Therapy) and in SOLID-TIMI 52 (Stabilization of Plaque Using Darapladib-Thrombolysis in Myocardial Infarction 52).^6,7^ We pooled results across trials by fixed-effect inverse-variance weighted meta-analysis (Supplementary Figures 2 and 3; see Supplementary text for details of the methods used).

### Statistical methods

We defined effect alleles as those associated with lower Lp-PLA_2_ activity and assumed an additive model. For participant-level data, we assessed associations of Lp-PLA_2_-lowering alleles with CHD using the genome-wide efficient mixed model analysis, an approach that models each genetic variant as a fixed-effect, but includes both fixed-effect and random-effects of genetic inheritance^30^ to account for population stratification and relatedness among participants (Supplementary Material). The four rare loss-of-function variants were tested jointly within each study by counting the number of loss-of-function alleles carried by each participant. Log odds ratios and standard errors were meta-analysed across studies using fixed-effect meta-analysis. For studies contributing only study-level data, we performed a similar test by conducting a combined burden test across studies using the R package seqMeta v1.2 (http://cran.r-project.org/web/packages/seqMeta/).

We calculated associations of Lp-PLA_2_-lowering alleles with soluble Lp-PLA_2_ activity and conventional risk factors using linear regression within each study, and then combined the regression coefficients using fixed-effect meta-analysis. When data were missing, we used information on rs1805018 as a proxy for Val279Phe and information on rs7756935 or rs3799277 as proxies for Val379Ala (Supplementary Material). To account for population stratification, we adjusted for the first principal component of ancestry (Supplementary Material). We calculated risk ratios for CHD with decrements in Lp-PLA_2_ activity, dividing the log transformed risk ratio and confidence interval (CI) by the effect on Lp-PLA_2_ activity of the instrument (i.e. the genetic variant).^31^ We investigated heterogeneity using the *I*^2^ statistic. We used Stata 13.1.

## Results

Of the 261,950 total participants in this analysis, we studied 195,715 individuals of European ancestry, 34,221 individuals of South Asian ancestry and 32,014 individuals of East Asian ancestry. In people of European or South Asian ancestry without CHD, the frequency of alleles in *PLA2G7* that lower Lp-PLA_2_ activity was 0.005% at c.109 + 2T > C, 0.04% at Arg82His, 0.04% at Val279Phe and 0.025% at Gln287Ter (i.e. in aggregate, 0.2% of the European or South Asian participants in the current study carried one of these loss-of-function alleles, although no one carried more than one of these variants), and about 80% at Val379Ala. In people of East Asian ancestry without CHD, the frequency of Val279Phe was about 15% and about 2% of the individuals were homozygous carriers of the 279Phe allele.

### Soluble Lp-PLA_2_ activity

Compared with non-carriers, homozygote carriers of the 279Phe allele had 94% lower Lp-PLA_2_ activity (*p* < 10^–300^). For each 279Phe allele inherited, Lp-PLA_2_ activity decreased by 45% (1.59 SD, 95% CI: 1.61–1.57; *p* < 10^–300^). In Europeans who inherited any one of the four rare Lp-PLA_2_ loss-of-function alleles, Lp-PLA_2_ activity decreased by 64% (2.25 SD, 2.68–1.83; *p* = 1.6 × 10^–25^). For each 379Ala allele inherited, Lp-PLA_2_ activity decreased by 2.7% (0.096 SD, 0.122–0.069; *p* = 1.9 × 10^–12^). By comparison, 160 mg once-daily darapladib reduced Lp-PLA_2_ activity by 65% (2.26 SD, 2.31–2.21; *p* < 10^–300^). Study-level estimates are provided in Supplementary Figure 2.

### Cardiovascular risk factors

**Figure 2.**
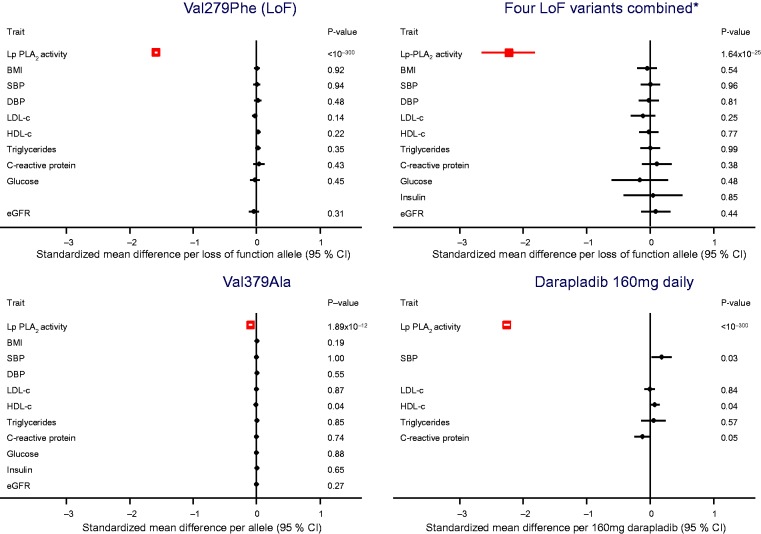
Mean per allele differences in Lp-PLA_2_ activity and cardiovascular risk factor levels by Lp-PLA_2_-lowering alleles or with darapladib 160 mg daily. To enable comparison of the magnitude of associations across several different markers, analyses were undertaken with standardized units of measurement for each marker. Associations are presented as per allele change in the biomarker expressed as standard deviations. Numbers of participants are provided in Table 1. Details of contributing studies are provided in Supplementary Material Tables 2 and 3 online. *Carriage of any of the four loss-of-function variants c.109+2T>C, Arg82His, Val279Phe, Gln287Ter. BMI: body-mass index; CI: confidence interval; DBP: diastolic blood pressure; eGFR: estimated glomerular filtration rate; HDL-c: high-density lipoprotein cholesterol; LDL: low-density lipoprotein cholesterol; LoF: loss-of-function; Lp-PLA_2_: lipoprotein associated phospholipase A_2_; SBP: systolic blood pressure

None of the Lp-PLA_2_-related variants we studied was significantly associated with values of LDL-cholesterol, HDL-cholesterol, triglycerides, systolic or diastolic blood pressure, body-mass index, estimated glomerular filtration rate, glucose, insulin and C-reactive protein (Figure 2). By comparison, in previous randomized placebo-controlled trials, darapladib did not significantly affect concentrations of LDL-cholesterol or log triglycerides, but could have slightly increased systolic blood pressure and HDL-cholesterol values and slightly decreased C-reactive protein concentration (Figure 2).

### Clinical CHD outcomes

**Figure 3.**
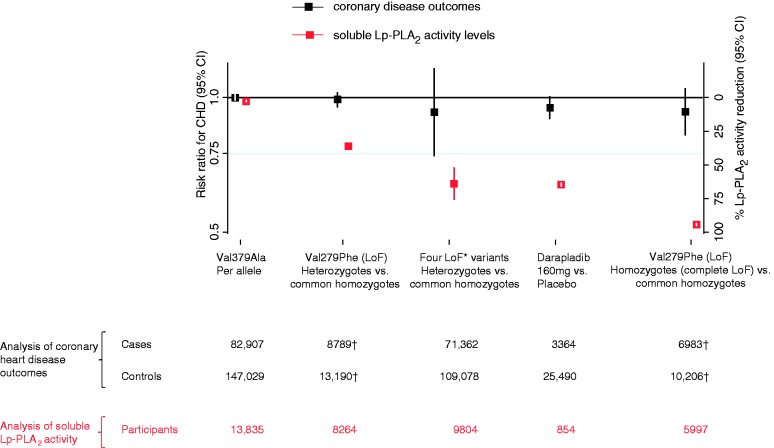
Association of Lp-PLA_2_-lowering alleles with Lp-PLA_2_ activity and CHD risk. Spectrum of functional alleles in *PLA2G7* and effects on Lp-PLA2 activity (red estimates) and coronary heart disease risk (black estimates); *Carriage of any of the four loss-of-function variants c.109+2T>C, Arg82His, Val279Phe, Gln287Ter. ^†^One study did not provide tabular data to enable calculation of CHD odds ratios in heterozygotes or homozygotes. Hence, numbers are less than those presented for the per allele analysis in Table 2. CHD: coronary heart disease; CI: confidence interval; LoF: loss-of-function; Lp-PLA_2_: lipoprotein associated phospholipase A_2_.

Compared with non-carriers, the odds ratio for CHD was 0.99 (0.95–1.03) in 279Phe heterozygotes, and 0.93 (0.82–1.05) in 279Phe homozygotes (i.e. nearly complete loss of Lp-PLA_2_ function: Figure 3). For each loss-of-function (279Phe) allele inherited, the odds ratio for CHD was 0.97 (0.91–1.02; *I*^2 ^= 30%; *p*_Heterogeneity_ = 0.2). In Europeans and South Asians who inherited one of the four rare Lp-PLA_2_-loss-of-function alleles, the odds ratio for CHD was 0.92 (0.74–1.16; *I*^2 ^= 0%; *p*_Heterogeneity_ = 0.8; Figure 3). For each 379Ala allele inherited, the odds ratio for CHD was 1.00 (0.98–1.02; *I*^2 ^= 0.0%; *p*_Heterogeneity_ = 0.5; Figure 3). Study-level results are provided in Supplementary Figure 3. In sensitivity analyses, odds ratios with each loss-of-function variant were similar to the odds ratio that combined information across the four loss-of-function variants we studied. There was no evidence of heterogeneity in odds ratios between European and South Asian ancestry populations (Supplementary Figure 4).

Genetic risk ratios for CHD per 65% lower Lp-PLA_2_ activity (i.e. the reduction achievable with darapladib treatment) were: 0.95 (0.88–1.03) with Val279Phe in East Asians; and 0.92 (0.74–1.16) with carriage of any one of the four rare variants studied in Europeans and South Asians; and 1.01 (0.68–1.51) with Val379Ala (Table 2). By comparison, the risk ratio for CHD with darapladib treatment (i.e. also per 65% lower Lp-PLA_2_ activity) was 0.95 (0.89–1.02; Table 2).

**Table 2.** Comparison on a common scale of human genetic and randomized trial evidence for Lp-PLA_2_ lowering and CHD.

	CHD patients	Controls	Risk ratio for CHD per 65% lower Lp-PLA_2_ activity (95% CI)
Genetically lowered Lp-PLA_2_			
Val279Phe (East Asian LoF variant)	10,088	15,199	0.95 (0.88–1.03)
Four loss-of-function variants^a^	71,362	109,078	0.92 (0.74–1.16)
Val379Ala	82,907	147,029	1.01 (0.68–1.51)
Pharmacologically lowered Lp-PLA_2_			
Darapladib	3364	25,490	0.95 (0.89–1.02)

**Endpoint**	**Val279Phe, loss-of-function variant common in East Asians**	**Four loss-of-function variants^a^, rare in Europeans and South Asians**		**Val379Ala, modest impact variant**				**Darapladib**
**Conventional risk factors** ^f^	12 East Asian studies^c^	Eight European or South Asian ancestry studies from the CHD Exome^+^ consortium^19-26^ ^d^		Eight European or South Asian ancestry studies from the CHD Exome^+^ consortium^19-26^ ^d^		Publicly available consortium data^e^		Five randomized clinical trials^6,7,39-41^ ^c^
BMI	17,898	76,584		51,201		126,142 from 46 studies from the GIANT consortium^42^		NA
Blood pressure	6705	72,450		71,256		69,245 from 29 studies from the ICBP consortium^43^		323
Lipids	17,643	76,826		55,431		94,311 from 46 studies from the GLGC consortium^44^		803
C-reactive protein	2914	40,484		41,442		66,185 from 15 studies from the CHARGE consortium^45^		848
Glycaemic traits	2914	9420		9408		46,186 from 21 studies from the MAGIC consortium^46^		NA
eGFR	4017	32,929		32,190		74,354 from 26 studies from the CKDGen consortium^47^		NA

Further detail on the individual studies is provided in Supplementary Tables 2 and 3 online.

a rs142974898 (c.109+2T>C), rs144983904 (Arg82His), rs76863441 (Val279Phe), rs140020965 (Gln287Ter); see also Figure 1(b) for further variant details.

b In genetic analysis, CHD was defined as myocardial infarction and other major coronary events (∼90% of cases) or angiographic stenosis only (∼10% of cases); see Supplementary Tables 2 and 3 for details. In the darapladib analysis CHD was defined as fatal coronary disease, non-fatal myocardial infarction or urgent revascularization for myocardial ischaemia.

c Summary/tabular data available (by study).

d Participant-level data available.

e Meta-analysis data available.

f See Supplementary Tables 2 and 3 for details on risk factor measurements.

BMI: body-mass index; C4D: Coronary Artery Disease Genetics consortium; CARDIoGRAM: Coronary ARtery DIsease Genome wide Replication and Meta-analysis; CHARGE: Cohorts for Heart and Aging Research in Genomic Epidemiology; CHD: coronary heart disease; CKDGen: Chronic Kidney Disease Genetics consortium; eGFR: estimated glomerular filtration rate; GIANT: Genetic Investigation of ANthropometric Traits consortium; GLGC: Global Lipids Genetics Consortium; ICBP: International Consortium for Blood Pressure; Lp-PLA_2_: lipoprotein associated phospholipase A_2_; MAGIC: Meta-Analyses of Glucose and Insulin-related traits Consortium; NA: data not available

## Discussion

In a large-scale analysis of human genetic data, we tested whether Lp-PLA_2_ enzyme activity is causally relevant to CHD by studying five functional alleles that produce widely differing (i.e. small, moderate or large) degrees of reduction in Lp-PLA_2_ activity. We found that none was related to CHD risk, suggesting that Lp-PLA_2_ enzyme activity is unlikely to be causally relevant to CHD, a conclusion concordant with results from two phase 3 trials of a pharmacological Lp-PLA_2_ enzyme inhibitor.

Three features of our study merit comment. First, we studied almost 20 times more CHD patients than the previous largest study of loss-of-function *PLA2G7* alleles, thereby providing the first robust genetic evaluation of effect sizes of Lp-PLA_2_ inhibition relevant to phase 3 trials such as relative risk reductions for CHD of 20%. For example, for the Val279Phe variant we had >99% power to detect a 20% risk reduction in CHD for a 65% genetic reduction in Lp-PLA2 activity (i.e. an effect on Lp-PLA2 activity similar to that achieved by darapladib).

Second, our study has provided the first investigation in CHD of a series of functional alleles that each reduce Lp-PLA_2_ function via different molecular mechanisms. Specifically, we studied five different Lp-PLA_2_-lowering alleles: three of the alleles were coding variants that produced different amino acid substitutions; two of the alleles produced protein truncations (one due to a nonsense mutation; the other due to a splice-site mutation). Because we observed null and broadly concordant findings for CHD risk across these alleles that each changed the enzyme in a different way (and to a different extent), we can more confidently conclude there is no material cause-and-effect relationship. By contrast, when the initial phase 3 trial of darapladib was launched in 2008, only two of the five alleles we studied had yet been identified: data on Val379Ala, a weak effect missense variant, were inconclusive because CHD studies were under-powered;^32^ data on Val279Phe, a loss-of-function variant, and CHD risk were sparse and restricted to East Asian populations.

A third feature was our study’s analysis of large-scale data from three different major ethnic groups: Europeans, South Asians and East Asians. This ethnic diversity enhanced the generalizability of our results.

Our study had potential limitations. To maximize comparability of CHD endpoints used in clinical trials with those used in human genetic studies, we restricted analysis of phase 3 darapladib trials to ‘major coronary events’ and principally focused in human genetic studies on the cognate endpoints of myocardial infarction or other major acute coronary events (which constituted ∼90% of the outcomes). Nevertheless, although the CHD definitions used in trials and genetic studies were similar, they were not identical.

It could be that cardioprotective benefits of Lp-PLA_2_ inhibition were obscured by pleiotropic effects of *PLA2G7* variants; for example, 279Phe is known to produce a misfolded version of Lp-PLA_2_ not secreted by cells, prompting suggestions that its carriage could produce ‘off-target’ effects such as increased cell death.^33,34^ However, because we found null associations between four other functional alleles in *PLA2G7* and CHD, each of which operates via a different molecular mechanism, it argues against this explanation. On the other hand, it is possible that darapladib may have additional effects beyond Lp-PLA_2_ inhibition. For example, darapladib may have had slight effects on CRP levels and systolic blood pressure, which we did not observe with the genetic variants.

Lifelong genetic reductions in Lp-PLA_2_ could result in compensatory responses that increase CHD risk. However, this explanation seems unlikely because it would require any such compensation to apply similarly across alleles that produce widely differing degrees of reduction in Lp-PLA_2_ activity. Furthermore, any such compensation could not operate through known cardiovascular mechanisms because we observed no associations between Lp-PLA_2_-lowering alleles and several established and emerging cardiovascular risk factors.

Soluble enzyme activity could be an imperfect indicator of the relevance of Lp-PLA_2_ to atherosclerotic plaques. However, for homozygote carriers of 279Phe, Lp-PLA_2_ activity should be almost abolished across all tissues. Finally, we studied life-long genetic reductions in Lp-PLA_2_ activity in relation to first-onset CHD outcomes rather than recurrent CHD, whereas darapladib trials studied recurrent coronary events in patients with stable or acute coronary disease.

The current data underscore the growing importance of human genetic approaches to enhance the efficiency of development of medicines by validating (or invalidating) novel drug targets.^35-38^ Specifically, despite beneficial effects of darapladib on surrogate markers (e.g. intravascular imaging) of coronary atherosclerosis in pre-clinical and clinical studies,^39-41^ these effects did not translate into reduced outcomes in the large phase 3 studies. Hence, human genetic studies may be useful in influencing prioritization of clinical outcome trials in the future.

Our results also illustrate how human genetic evidence can assist interpretation of observational epidemiological data. For example, we found that functional alleles in *PLA2G7* do not alter levels of pro-atherogenic lipids (e.g. LDL-cholesterol), suggesting that such pro-atherogenic lipids do not mediate associations between Lp-PLA_2_ activity and CHD and supporting the need to adjust epidemiological associations of Lp-PLA_2_ activity with CHD risk for pro-atherogenic lipids (an approach which yields results consistent with non-causality).^3^

In summary, we found that none of a series of Lp-PLA_2_–lowering alleles was related to CHD risk, suggesting that Lp-PLA_2_ is unlikely to be a causal risk factor in CHD.

## Supplementary Material

Supplementary material
