# Supplementary material for: Genetic invalidation of Lp-PLA2 as a therapeutic target: Large-scale study of five functional Lp-PLA2-lowering alleles
Source: Eur J Prev Cardiol. 2016 Dec 8;24(5):492–504. doi: 10.1177/2047487316682186 (PMC5460752; doi:10.1177/2047487316682186)
Supplement: Supplementary material [file CPR682186_supplementary_material.pdf]

SUPPLEMENT: Genetic invalidation of Lp-PLA$_2$ as a therapeutic target: large-scale study of five functional Lp-PLA2 lowering alleles

Systematic review of East Asian studies

We searched the Medline electronic library on 04/07/2014 by combining the following terms related to the *PLA2G7* genetic variant (**see eFigure 1**), without language restriction:

(Lp-PLA2 OR LpPLA2 OR Lp-PLA(2) OR 1-alkyl-2-acetylglycerophosphocholine esterase OR PAFAH OR PAF-AH OR PLA2G7 OR platelet activating factor acetylhydrolase OR lipoprotein associated phospholipase A2)

AND

(gene OR genes OR polymorph* OR allel* OR phenotyp* OR SNP OR chromosom* OR variant* OR mutat* OR locus OR loci OR Genes [Mesh] OR Polymorphism, genetic [Mesh] OR RFLP OR V279F OR PLA2G7 or rs16875954)

Titles, abstracts and full text versions of identified articles were reviewed independently by at least two of three investigators (JG, AT, DFF). Studies were included if they reported on, or allowed calculation of, the association of the loss of function variant V279F with coronary heart disease (provided myocardial infarction was part of the outcome definition), or the risk factors in healthy individuals. Additionally, we searched the NIHR GWAS catalogue (https://www.genome.gov/26525384; accessed 04/07/2014) to identify additional GWAS studies of coronary disease in East Asians. eFigure 1 describes the selection process, which identified 16 independent studies overall that contributed data.

Systematic review of randomized controlled darapladib trials

We searched the Medline electronic library on 23/02/2015 for the term "darapladib" without language restriction. Titles, abstracts and full text versions of identified articles were reviewed independently by two investigators (JG, DFF). The search strategy identified 75 publications in total. Five of these publications reported on the results of five randomized placebo controlled clinical trials of darapladib. Three of those trials reported on Lp-PLA$_2$ activity and cardiovascular risk factors, two trials reported on coronary heart disease endpoints.

Proxy variants

In cases where information on the genetic variants was not available, we identified suitable proxy variants for the relevant population using 1000 Genomes, Phase I release data. For the modest impact variant rs1051931, which is common in Europeans, we identified suitable proxy variants in Europeans: rs7756935 (r2=1.00, D'=1.00) and rs3799277 (r2=0.96, D'=1.00). For the loss of function variant rs76863441 (previously rs16874954), common in East Asians, we identified rs1805018 as a suitable proxy variant in Japanese populations (r2=0.94, D'=1.0).

Adjustment for principal components in genetic analyses

Principal component analysis (PCA) was performed (separately for Europeans and South Asians) in the component studies of the CHD Exome+ Consortium to identify ethnic outliers. Following standard sample and variant QC, further stringent variant QC (call rate>99% and Hardy-Weinberg p-value<$1x10^{-4}$) was performed on common variants (minor allele frequency>0.05), followed by stepwise pruning until no residual linkage disequilbrium (r^2>0.2) was observed. We further excluded variants in known coronary disease loci, leaving 19,256 variants within the Europeans and 17,968 variants within the South Asians for the PCA. PCA was then performed on a standardised genotype matrix created using the Singular Value Decomposition (SVD) function implemented in the R package 'irlba'. 219 Europeans and 10 South Asians who were ancestral outliers, defined as being at least 3

standard deviations away from the cluster mean, were excluded. The PCA was rerun after excluding these samples to obtain principal components that could be used to account for population substructure. The first principal component was included in the statistical model for each ancestral group.

ACKNOWLEDGEMENTS

We thank Dr. Michael Sweeting and Dr. Angela Wood for helpful comments on an earlier version of this manuscript, and Dr. Richard Gibbs and Dr. Dawn Waterworth for contributing tabular data.

The MORGAM consortium: Dr. Salomaa was supported by the Finnish Foundation for Cardiovascular Research. This work has been sustained by the MORGAM Project's recent funding: European Union FP 7 projects ENGAGE (HEALTH-F4-2007-201413), CHANCES (HEALTH-F3-2010-242244) and BiomarCaRE (278913). This has supported central coordination, workshops and part of the activities of the The MORGAM Data Centre, at THL in Helsinki, Finland. MORGAM Participating Centres are funded by regional and national governments, research councils, charities, and other local sources. The PRIME Study was supported between 1992 and 1997 by grants from an agreement between the Institut National de la Santé et de la Recherche Médicale (Inserm) and the Merck, Sharp and Dohme-Chibret Laboratory. The KORA research platform (KORA, Cooperative Research in the Region of Augsburg) was initiated and financed by the Helmholtz Zentrum München - German Research Center for Environmental Health, which is funded by the German Federal Ministry of Education and Research and by the State of Bavaria. Furthermore, KORA research was supported within the Munich Center of Health Sciences (MC Health), Ludwig-Maximilians-Universität, as part of LMUinnovativ. MORGAM Italy: EPIMED Research Center, Department of Clinical and Experimental Medicine, University of Insubria, Varese: M. Ferrario (principal investigator), G. Veronesi, F. Gianfagna. University of Milano-Bicocca, Monza, Italy, Giancarlo Cesana, Paolo Brambilla and Stefano Signorini. This study was supported by the Health Administration of Regione Lombardia [grant numbers 9783/1986, 41795/1993, 31737/1997,17155/2004 and 10800/2009].

The ARIC study: The ARIC study was supported by a sponsored project (RC2HL102419) from the National Heart, Lung, and Blood Institute (NHLBI), contracts (HHSN268201100005C, HHSN268201100006C, HHSN268201100007C, HHSN268201100008C, HHSN268201100009C, HHSN268201100010C, HHSN268201100011C, and HHSN268201100012C) with the NHLBI, and a grant (U54 HG003273) from the National Human Genome Research Institute.

Drs Kato and Takeuchi thank Dr. Eitaro Nakashima, Dr. Tomohiro Katsuya, and Dr. Masato Isono for their assistance in collecting the DNA samples and the accompanying clinical information and in genotyping the SNPs.

Drs Kimura, Sasaoka thank Dr. Shigeru Hohda (Department of Molecular Pathogenesis, Medical Research Institute, Tokyo Meidcal and Dental University) for his contribution in genotyping.

CKDGen consortium: Mathias Gorski was supported by the Else-Kröner-Fresenius-Stiftung (2012_A147)

ICBP: Patricia B Munroe: This work forms part of the research programme of the NIHR Cardiovascular Biomedical Research Unit at Barts; George B Ehret is supported by the University of Geneva, NHLBI (RO1HL086694), The Swiss National Foundation (FN 33CM30-124087), and the Fondation pour Recherches Medicales and the GeCor Foundation.

SUPPLEMENTARY REFERENCES

1. Randall JC, Winkler TW, Kutalik Z et al. Sex-stratified genome-wide association studies including 270,000 individuals show sexual dimorphism in genetic loci for anthropometric traits. PLoS Genet 2013;9(6):e1003500.

2. Ehret GB, Munroe PB, Rice KM et al. Genetic variants in novel pathways influence blood pressure and cardiovascular disease risk. Nature 2011;478(7367):103-109.

3. Serruys PW, Garcia-Garcia HM, Buszman P et al. Effects of the direct lipoprotein-associated phospholipase A(2) inhibitor darapladib on human coronary atherosclerotic plaque. Circulation 2008;118(11):1172-1182.

4. Mohler ER, III, Ballantyne CM, Davidson MH et al. The effect of darapladib on plasma lipoprotein-associated phospholipase A2 activity and cardiovascular biomarkers in patients with stable coronary heart disease or coronary heart disease risk equivalent: the results of a multicenter, randomized, double-blind, placebo-controlled study. J Am Coll Cardiol 2008;51(17):1632-1641.

5. Daida H, Iwase T, Yagi S, Ando H, Nakajima H. Effect of darapladib on plasma lipoprotein-associated phospholipase A2 activity in Japanese dyslipidemic patients, with exploratory analysis of a PLA2G7 gene polymorphism of Val279Phe. Circ J 2013;77(6):1518-1525.

6. O'Donoghue ML, Braunwald E, White HD et al. Effect of darapladib on major coronary events after an acute coronary syndrome: the SOLID-TIMI 52 randomized clinical trial. JAMA 2014;312(10):1006-1015.

7. White HD, Held C, Stewart R et al. Darapladib for preventing ischemic events in stable coronary heart disease. N Engl J Med 2014;370(18):1702-1711.

8. Willer CJ, Schmidt EM, Sengupta S et al. Discovery and refinement of loci associated with lipid levels. Nat Genet 2013;45(11):1274-1283.

9. Dehghan A, Dupuis J, Barbalic M et al. Meta-analysis of genome-wide association studies in >80 000 subjects identifies multiple loci for C-reactive protein levels. Circulation 2011;123(7):731-738.

10. Dupuis J, Langenberg C, Prokopenko I et al. New genetic loci implicated in fasting glucose homeostasis and their impact on type 2 diabetes risk. Nat Genet 2010;42(2):105-116.

11. Pattaro C, Kottgen A, Teumer A et al. Genome-wide association and functional follow-up reveals new loci for kidney function. PLoS Genet 2012;8(3):e1002584.

Supplementary figures and tables

eFigure1: Search strategy and study flow diagram for systematic review and meta-analysis of V279F (rs76863441) and cardiovascular disease

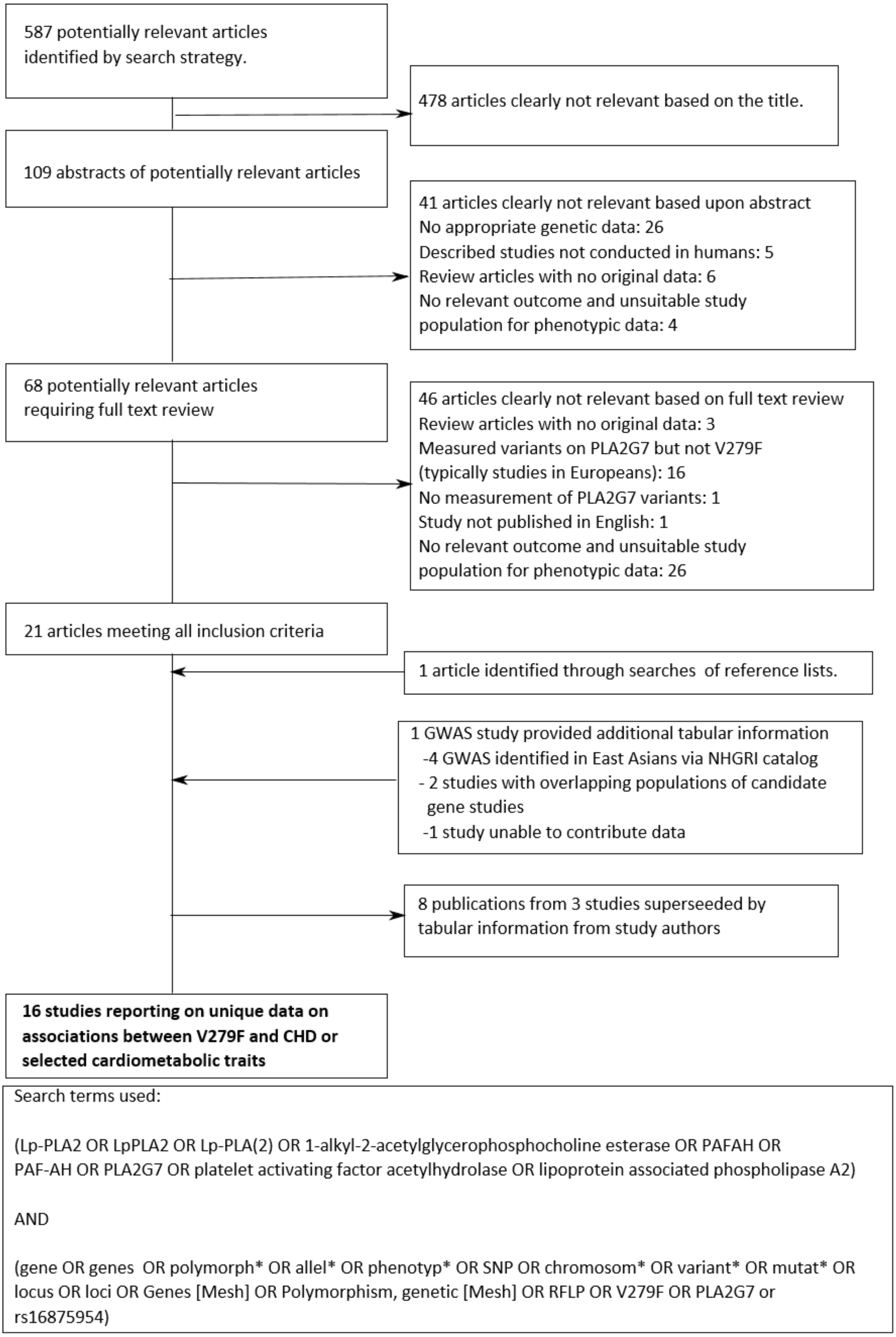

eFigure 2: Meta-analysis of associations of genetic variants in *PLA2G7* or darapladib with Lp-PLA2 activity.

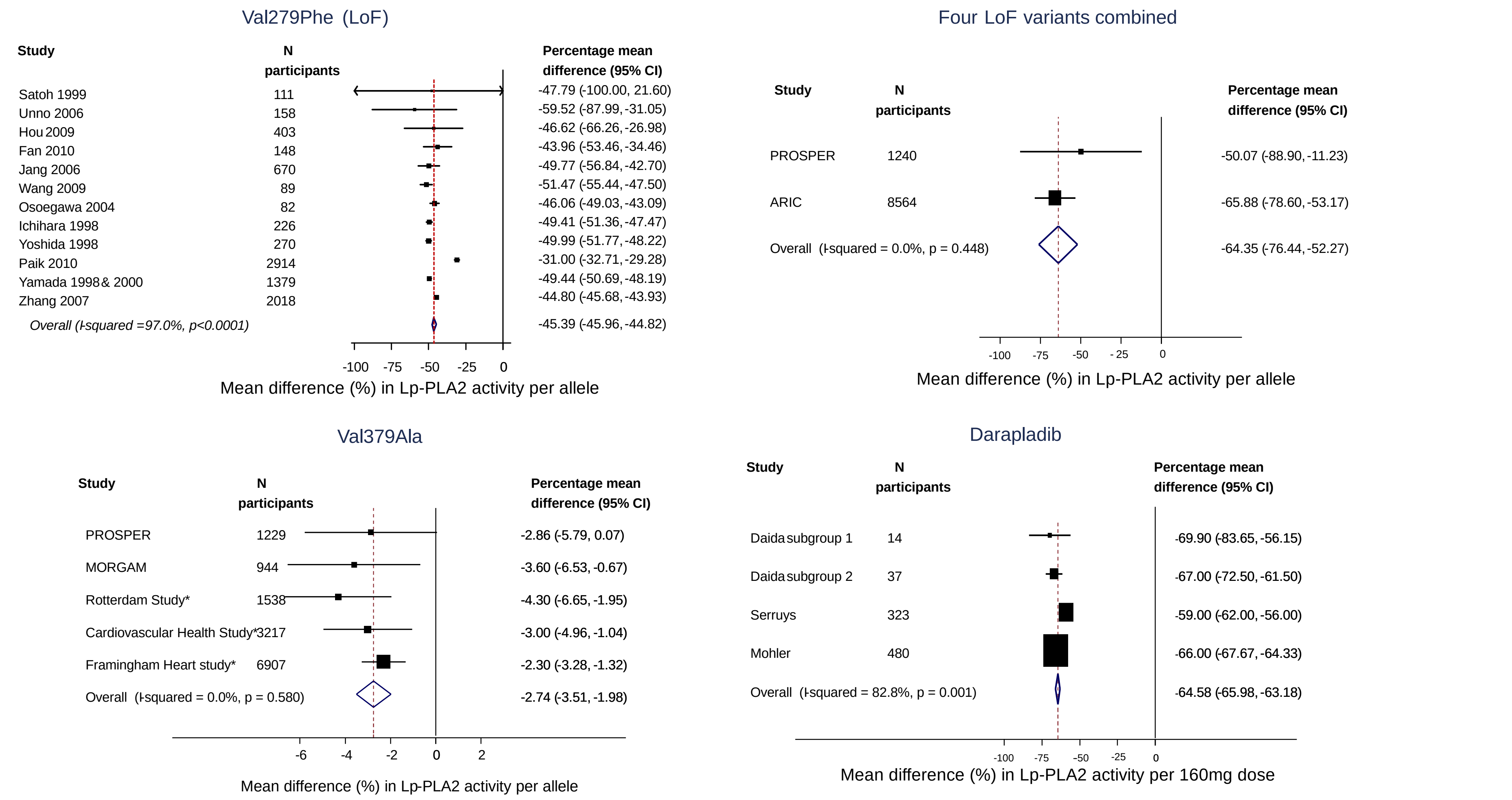

* proxy variant rs7756935 (r^2=1.00); MORGAM = MONICA, Risk, Genetics, Archiving, and Monograph; PROMIS = Pakistan Risk of Myocardial Infarction Study; PROSPER = Pravastatin in elderly individuals at risk of vascular disease trial

+Estimate of difference in Lp-PLA2 activity per rare V279F allele using a random-effects meta-analysis with inverse-variance weighting is -46.3% (95% CI: -50.5% to -42.0%)

eFigure 3: Meta-analysis of associations of genetic variants in *PLA2G7* or darapladib with coronary heart disease risk.

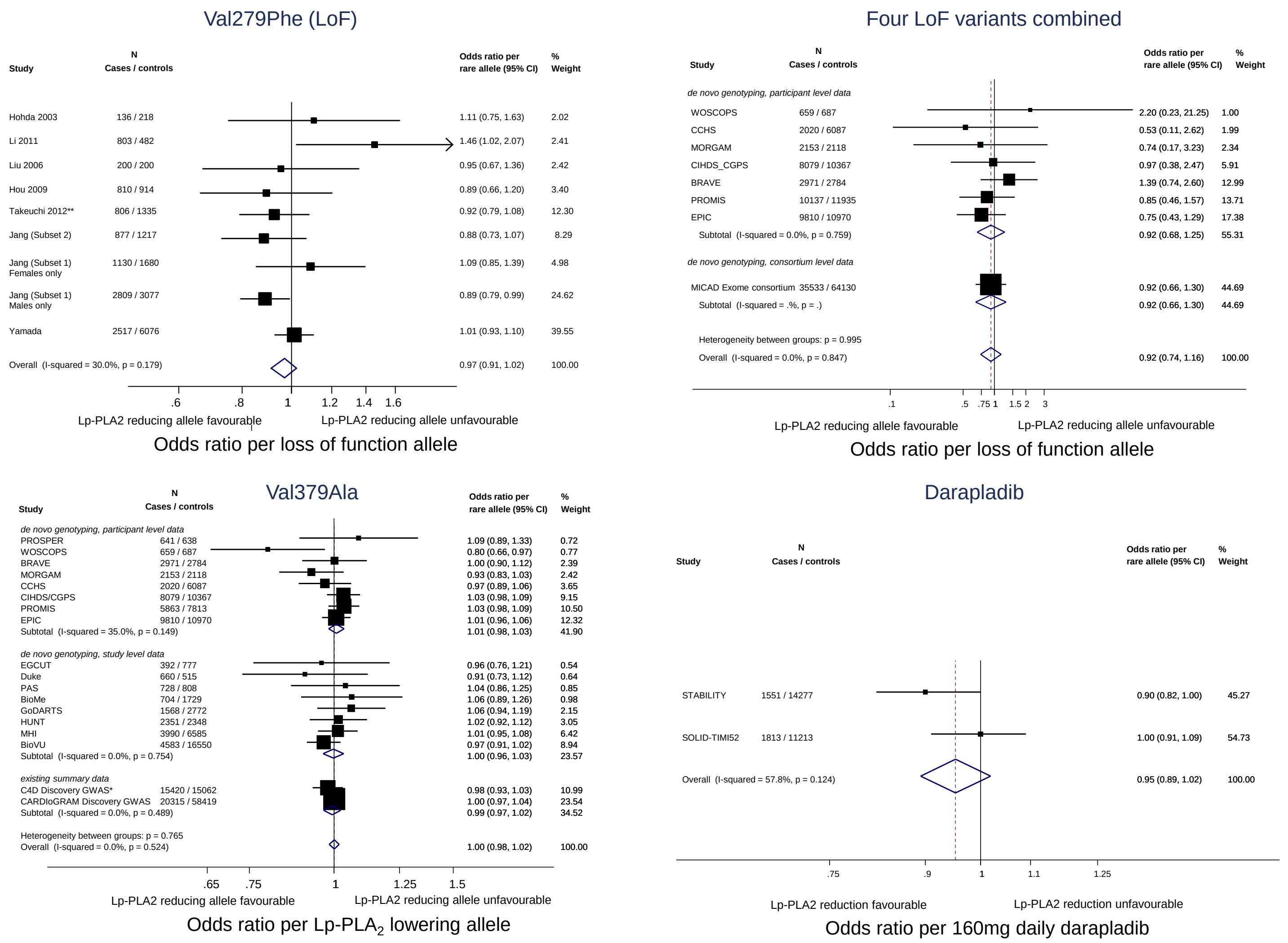

BRAVE = Bangladesh Risk of Acute Vascular Events Study; C4D = the Coronary Artery Disease Genetics consortium; CARDIoGRAM = the transatlantic Coronary Artery Disease Genome-wide Replication and Meta-analysis consortium; CCHS = Copenhagen City Heart Study; CHS= Cardiovascular Health Study; CGPS = Copenhagen General Population Study; CIHDS = Copenhagen Ischaemic Heart Disease Study; EPIC = European Prospective Investigation into Cancer and Nutrition Study; MORGAM = MONICA, Risk, Genetics, Archiving, and Monograph; PROMIS = Pakistan Risk of Myocardial Infarction Study; PROSPER = Pravastatin in elderly individuals at risk of vascular disease trial; WOSCOPS = West of Scotland Coronary Prevention Study; * proxy variant rs3799277 (r^2=0.96 with rs1051931 in Europeans); ** proxy variant rs1805018 (r^2=0.94 with rs76863441 in Japanese). CHD estimate from darapladib clinical trials uses the endpoint fatal CHD, MI or urgent revascularisation (primary endpoint for SOLID-TIMI-52, and pre-specified secondary endpoint for STABILITY).

eFigure 4: Individual and combined estimates for loss of function variants in Europeans and South Asians

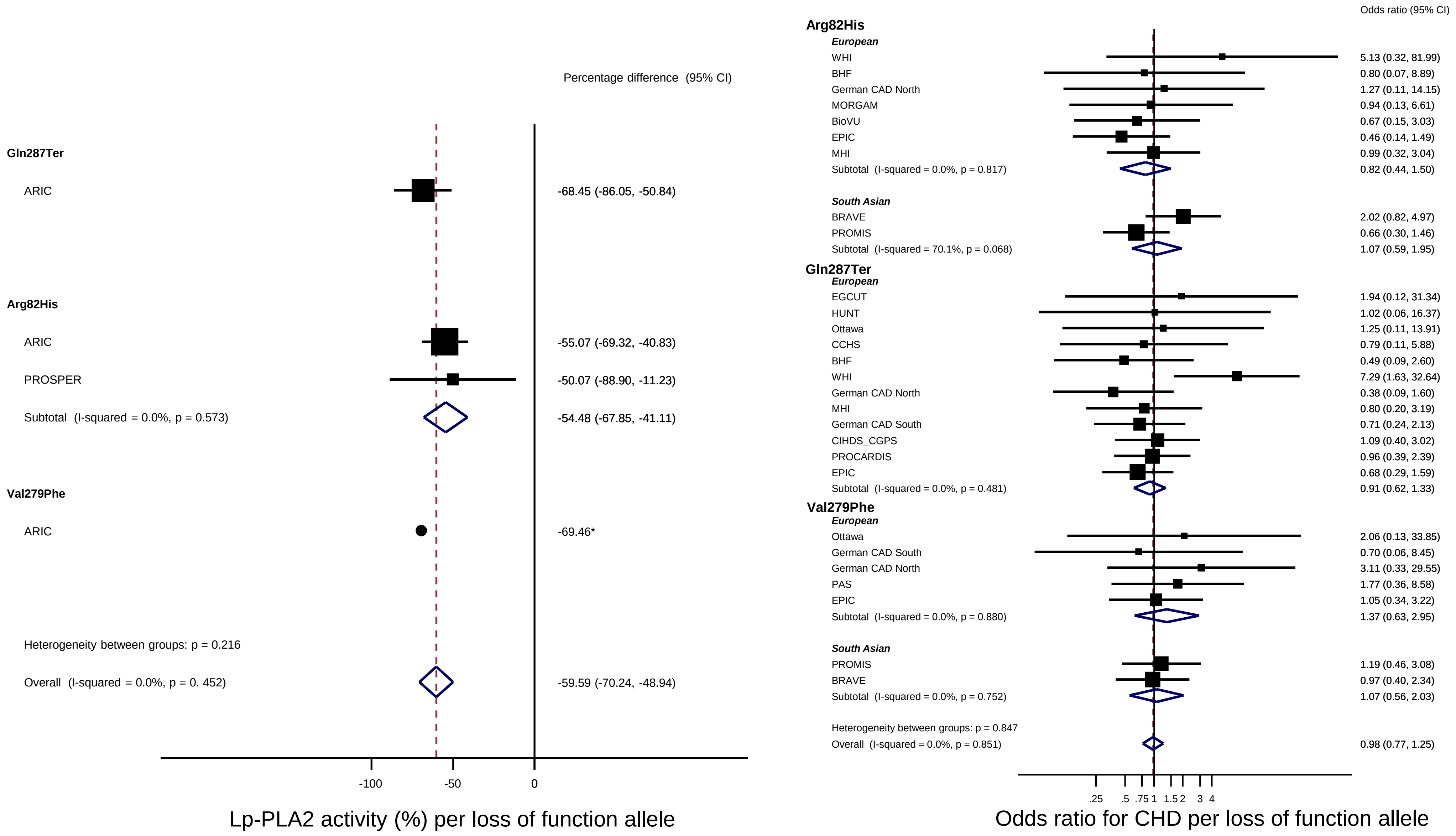

*Estimate based on one carrier of Val279Phe, hence the confidence interval has been omitted. There were no individuals with Lp-PLA2 activity levels and the splice donor loss of function variant c.109+2T>C (rs142974898) measured. With an allele frequency of 0.01% (~8 times less common than the other loss of function variants), this variant was too rare for individual variant testing. The variant contributed, however, to the aggregated test over all four loss of function variants presented in the main manuscript

eTable 1: Loss of function variants in *PLA2G7* and annotation

Protein change or context*	Rsid (if available)	Observed in 1KG or ESP†	Primary source	Reference for *in vivo* or *in vitro* evidence	Selected for present study
69 D>G	rs201315851	Neither	Re-sequencing study	Song Pharmacogenomics 2011	No
82 R>H	rs144983904	1KG, ESP	Re-sequencing study	Song Pharmacogenomics 2011	Yes
134 W>STOP	rs200454121	Neither	Re-sequencing study	Song Pharmacogenomics 2011	No
181 D>G	NA	Neither	Re-sequencing study	Song Pharmacogenomics 2011	No
273 S>A	NA	Neither	UniProt	Tjoelker LW, JBC 1995	No
273 S>F	NA	Neither	Re-sequencing study	Song Pharmacogenomics 2011	No
279 V>F	rs76863441 / rs16874954	1KG	Uniprot	Several	Yes
281 Q>R	rs201256712	Neither	Uniprot	Yamada Y, Biochem Biophys Res Commun 1997	No
283 L>P	rs200303358	Neither	Re-sequencing study	Song Pharmacogenomics 2011	No
286 D>A	NA	Neither	UniProt	Tjoelker LW, JBC 1995	No
286 D>N	NA	Neither	Uniprot	Tjoelker LW, JBC 1995	No
287 Q>STOP	rs140020965	1KG, ESP	ExAC consortium		Yes
296 D>A	NA	Neither	Uniprot	Tjoelker LW, JBC 1995	No
296 D>N	NA	Neither	Uniprot	Tjoelker LW, JBC 1995	No
351 H>A	NA	Neither	Uniprot	Tjoelker LW, JBC 1995	No
Splice donor variant	rs142974898	1KG	ExAC consortium		Yes

* refers to the canonical transcript ENST00000274793 †(1KG = observed in 1000 Genomes project; ESP = observed in Exome sequencing project). Only loss of function variants reported in either 1KG or ESP were included in this study (entries shaded in light blue).

143 **eTable 2:** Summary of Caucasian & South Asian studies/consortia

Endpoint / phenotype	Type of data	Study / consortium	Endpoint definition	N cases	N controls	Reference (PMID)
Coronary disease	*De-novo* genotyping	BRAVE	Confirmed MI meeting all of the following criteria: i) presented within 24 hours of the onset of sustained clinical symptoms suggestive of MI lasting longer than 20 minutes, including chest pain and breathlessness; ii) had ECG changes indicative of MI (new pathologic Q waves, at least 1 mm ST elevation in any 2 or more contiguous limb leads or a new left bundle branch block, or new persistent ST-T wave changes diagnostic of a non-Q wave MI) with a subsequent confirmation by troponin-I measurements; and iii) had no previous cardiovascular diseases; defined as self-reported history of angina, MI, coronary revascularisation, transient ischaemic attack, stroke or evidence of CHD on prior ECG or in other medical records.	2971	2784	25930055
	De-novo genotyping	CCHS	MI or major coronary event defined according to IHD (World Health Organization International Classification of Diseases-Eighth Revision, codes 410 to 414; International Classification of Diseases-Tenth Revision, codes I20 to I25) ascertained by reviewing all hospital admissions and diagnoses entered in the national Danish Patient Registry and all causes of death entered in the national Danish Causes of Death Registry.	2020	6087	19509380
	De-novo genotyping	CIHDS/CGPS	WHO-ICD 8 codes 410 to 414 or ICD 10 codes I20 to I25. Diagnosis of acute coronary syndrome and stenosis or atherosclerosis on coronary angiography and/or positive results on exercise electrocardiography.	8079	10,367	23265341; 17635890
	De-novo genotyping	EPIC	Incident CHD cases defined as fatal and non-fatal MI and other major acute coronary events, according to ICD-10 codes I20-I25. All centres have recorded cause-specific mortality through mortality registries and/or active follow-up, and have ascertained and validated incident fatal and non-fatal CHD through a combination of methods (eg, morbidity registers, general practice records, MONICA registries, self-report, clinical records).	9810	10,970	17295097
	De-novo genotyping	MORGAM	Incident definite or possible MI or coronary death, or unstable angina during follow-up, coronary revascularization during follow-up, documented MI at baseline, or an unclassifiable coronary death during follow-up	2153	2118	15561751
	De-novo genotyping	PROMIS	Confirmed MI meeting all of the following criteria: i) presented within 24 hours of the onset of sustained clinical symptoms suggestive of MI lasting longer than 20 minutes, including chest pain and breathlessness; ii) had ECG changes indicative of MI (new pathologic Q waves, at least 1 mm ST elevation in any 2 or more contiguous limb leads or a new left bundle branch block, or new persistent ST-T wave changes diagnostic of a non-Q wave MI) with a subsequent confirmation by troponin-I measurements; and iii) had no previous cardiovascular diseases; defined as self-reported history of angina, MI, coronary revascularisation, transient ischaemic attack, stroke or evidence of CHD on prior ECG or in other medical records.	10,137	11,935	19404752
	De-novo genotyping	PROSPER	Death from coronary heart disease or nonfatal MI.	641	638	12457784
	De-novo genotyping	WOSCOPS	Death from coronary heart disease or nonfatal MI.	659	687	7566020
	Summary	C4D	MI and other major coronary events (~90% of	11,146	10,940	21378988

	data, published		cases); angiographic stenosis only (~10% of cases)			
	Summary data, published	CARDIoGRAM	MI and other major coronary events (~90% of cases); angiographic stenosis only (~10% of cases)	20,315	58,415	21378990
Lp-PLA2 activity	Summary data, published	CHARGE consortium, composed of: Cardiovascular Health Study, Framingham Heart Study and Rotterdam Study	Validated colourimetric or radioactive assays (diaDexus CAM Kit, diaDexus, Inc., San Francisco, CA, USA or Perkin Elmer Life Sciences, Inc., Waltham, MA, USA)		12,113	22003152
	Tabular data, published	ARIC	Automated Colorimetric Activity Method assay (diaDexus Inc., South San Francisco, CA) using a Beckman Coulter (Olympus) AU400e autoanalyzer		8564	25587968
	De-novo genotyping	MORGAM (FINRISK component)	Colorimetric Activity Method assay, Diadexus, Inc., San Francisco, CA		944	15561751
	De-novo genotyping	PROSPER	Colorimetric Activity Method assay, Diadexus, Inc., San Francisco, CA		1229	20005516
LDL-C	Summary data, published	GLGC	Validated commercially available assays		89,888	24097068
	De-novo genotyping	BRAVE	Validated enzymatic assay (Roche Diagnostics)		5737	25930055
	De-novo genotyping	CCHS	Validated colorimetric assay		8056	19509380
	De-novo genotyping	CIHDS/CGPS	Validated colorimetric assay		18021	23265341; 17635890
	De-novo genotyping	EPIC	Validated assay (Roche Diagnostics)		17886	17295097
	De-novo genotyping	PROMIS	Validated enzymatic assay (Roche Diagnostics)		20885	19404752
	De-novo genotyping	PROSPER	Calculated using Friedewald formula		1268	12457784
	De-novo genotyping	WOSCOPS	Calculated using Friedewald formula		1337	7566020
HDL-C	Summary data, published	GLGC	Validated commercially available assays		94,311	24097068
	De-novo genotyping	BRAVE	Validated enzymatic assay (Roche Diagnostics)		5739	25930055
	De-novo genotyping	CCHS	Validated colorimetric assay		8096	19509380
	De-novo genotyping	CIHDS/CGPS	Validated colorimetric assay		18072	23265341; 17635890
	De-novo genotyping	EPIC	Validated assay (Roche Diagnostics)		18238	17295097
	De-novo genotyping	MORGAM	Validated enzymatic assay after isolation of HDL		4269	15561751
	De-novo genotyping	PROMIS	Validated enzymatic assay (Roche Diagnostics)		20919	19404752
	De-novo genotyping	PROSPER	Validated assay at the Centre for Disease Control certified central lipoprotein laboratory in Glasgow.		1268	12457784
	De-novo genotyping	WOSCOPS	Validated assay at the Centre for Disease Control certified central lipoprotein laboratory in Glasgow.		1337	7566020
Triglycerides	Summary data, published	GLGC	Validated commercially available assays		91,013	24097068
	De-novo genotyping	BRAVE	Validated enzymatic assay (Roche Diagnostics)		5738	
	De-novo genotyping	CCHS	Validated colorimetric assay		8068	19509380
	De-novo genotyping	CIHDS/CGPS	Validated colorimetric assay		18120	23265341; 17635890
	De-novo genotyping	EPIC	Validated assay (Roche Diagnostics)		18238	17295097

	De-novo genotyping	MORGAM	Validated assays	2316	15561751
	De-novo genotyping	PROMIS	Validated enzymatic assay (Roche Diagnostics)	20935	19404752
	De-novo genotyping	PROSPER	Validated assay at the Centre for Disease Control certified central lipoprotein laboratory in Glasgow.	1268	12457784
	De-novo genotyping	WOSCOPS	Validated assay at the Centre for Disease Control certified central lipoprotein laboratory in Glasgow.	1337	7566020
BMI	Summary data, published	GIANT	Predominantly measured (few individual studies with self-report)	126,142	23754948
	De-novo genotyping	BRAVE	Measured	5266	25930055
	De-novo genotyping	CCHS	Measured	8082	19509380
	De-novo genotyping	CIHDS/CGPS	Measured	14254	23265341; 17635890
	De-novo genotyping	EPIC	Measured or self-report	21117	17295097
	De-novo genotyping	MORGAM	Measured	4264	15561751
	De-novo genotyping	PROMIS	Measured	20994	19404752
	De-novo genotyping	PROSPER	Measured	1268	12457784
	De-novo genotyping	WOSCOPS	Measured	1337	7566020
Diastolic blood pressure	Summary data, published	ICBP	Validated methods (eg, mercury sphygmomanometer, automated blood pressure monitoring systems)	69,239	21909115
	De-novo genotyping	BRAVE	Standard sphygmomanometer	5250	25930055
	De-novo genotyping	CCHS	Standard sphygmomanometer	8070	19509380
	De-novo genotyping	CIHDS/CGPS	Standard sphygmomanometer	13215	23265341; 17635890
	De-novo genotyping	EPIC	Standard sphygmomanometer	15674	17295097
	De-novo genotyping	MORGAM	Simple mercury sphygmomanometer, random zero sphygmomanometer, or automated device	5755	15561751
	De-novo genotyping	PROMIS	Standard sphygmomanometer	20676	19404752
	De-novo genotyping	PROSPER	Standard mercury sphygmomanometer	1264	12457784
	De-novo genotyping	WOSCOPS	Standard mercury sphygmomanometer	1337	7566020
Systolic blood pressure	Summary data, published	ICBP	Validated methods (eg, mercury sphygmomanometer, automated blood pressure monitoring systems)	69,245	21909115
	De-novo genotyping	BRAVE	Standard sphygmomanometer	5250	25930055
	De-novo genotyping	CCHS	Standard sphygmomanometer	8070	19509380
	De-novo genotyping	CIHDS/CGPS	Standard sphygmomanometer	13218	23265341; 17635890
	De-novo genotyping	EPIC	Standard sphygmomanometer	15676	17295097
	De-novo genotyping	MORGAM	Simple mercury sphygmomanometer, random zero sphygmomanometer, or automated device	5755	15561751
	De-novo genotyping	PROMIS	Standard sphygmomanometer	20686	19404752
	De-novo genotyping	PROSPER	Standard mercury sphygmomanometer	1264	12457784
	De-novo genotyping	WOSCOPS	Standard mercury sphygmomanometer	1337	7566020
Insulin	Summary data, published	MAGIC	Validated commercially available assays	38,238	20081858

	De-novo genotyping	PROMIS	Validated assay	8073	19404752
	De-novo genotyping	PROSPER	Validated assay, Mercodia; Diagenics, Milton Keynes, UK	1236	12457784
Fasting glucose	Summary data, published	MAGIC	Validated commercially available assays	46,186	20081858
	De-novo genotyping	EPIC	Validated assay (Roche Diagnostics)	2606	17295097
	De-novo genotyping	PROMIS	Validated assay (Roche Diagnostics)	4234	19404752
	De-novo genotyping	PROSPER	Validated assay	1222	12457784
	De-novo genotyping	WOSCOPS	Validated assay	1346	7566020
eGFR	Summary data, published	CKDGen	Serum creatinine calibrated to the US nationally representative National Health and Nutrition Examination Study (NHANES) standards. eGFR was estimated using the four-variable MDRD Study equation.	74,354	22479191
	De-novo genotyping	EPIC	Serum creatinine (Roche Diagnostics). eGFR was estimated using the four-variable MDRD Study equation.	17133	17295097
	De-novo genotyping	PROMIS	Creatinine via Jaffe method. eGFR was estimated using the four-variable MDRD Study equation.	12444	19404752
	De-novo genotyping	PROSPER	Creatinine via Jaffe method. eGFR was estimated using the four-variable MDRD Study equation.	1267	12457784
	De-novo genotyping	WOSCOPS	Creatinine via Jaffe method. eGFR was estimated using the four-variable MDRD Study equation.	1346	7566020
C-reactive protein	Summary data, published	CHARGE	Validated ELISA or similar methods	66,185	21300955
	De-novo genotyping	CCHS	Turbidimetry (Dako, Glostrup, Denmark)	7349	19509380
	De-novo genotyping	CIHDS/CGPS	Nephelometry (Dade Behring, Deerfield, Ill)	11,425	23265341; 17635890
	De-novo genotyping	EPIC	Validated assay (Roche Diagnostics)	18231	17295097
	De-novo genotyping	MORGAM	Validated assays	971	15561751
	De-novo genotyping	PROMIS	Validated assay	1017	19404752
	De-novo genotyping	PROSPER	Turbidimetry (Roche UK, Welwyn Garden City, UK)	1239	12457784
	De-novo genotyping	WOSCOPS	Standardized in-house ELISA	1209	7566020

BRAVE = Bangladesh Risk of Acute Vascular Events Study; C4D = the Coronary Artery Disease Genetics consortium; CARDIoGRAM = the transatlantic Coronary Artery Disease Genome-wide Replication and Meta-analysis consortium; CCHS = Copenhagen City Heart Study; CHS= Cardiovascular Health Study; CGPS = Copenhagen General Population Study; CIHDS = Copenhagen Ischaemic Heart Disease Study; ELISA = Enzyme-linked immunosorbent assay; EPIC = European Prospective Investigation into Cancer and Nutrition Study; MORGAM = MONICA Risk, Genetics, Archiving and Monograph (subcohorts include: ATBC, Augsburg, Brianza, FINRISK, PRIME-Belfast, PRIME-Lille, PRIME-Strasbourg, PRIME-Toulouse); PROMIS = Pakistan Risk of Myocardial Infarction Study; PROSPER = Pravastatin in elderly individuals at risk of vascular disease trial; WOSCOPS = West of Scotland Coronary Prevention Study; Details for assessment methods used in the MORGAM cohort are available at: http://www.thl.fi/publications/morgam/qa/contents.htm

eTable 3 : Characteristics of participants from the Exome+ consortium by case-status

	South Asian				European											
	**BRAVE**		**PROMIS**		**CCHS**		**CGPS/CIHDS**		**EPIC-CVD**		**MORGAM**		**PROSPER**		**WOSCOPS**	
	Cases	Controls	Cases	Controls	Cases	Controls	Cases	Controls	Cases	Controls	Cases	Controls	Cases	Controls	Cases	Controls
N	2971	2874	10,137	11,935	2020	6087	8079	10,367	9810	10,970	2153	2118	641	638	659	687
Male (n (%))	2640 (88.9)	2467 (88.6)	8524 (84.1)	9054 (75.9)	1040 (51.5)	2553 (41.9)	5570 (68.9)	4706 (45.4)	6013 (61.3)	3779 (34.5)	1899 (89.2)	1753 (82.8)	403 (62.9)	305 (47.8)	659 (100)	687 (100)
Age (years)	52.1 (10.5)	50.2 (10.1)	53.7 (10.2)	56.2 (9.1)	65.7 (10.9)	55.3 (15.4)	63.8 (10.9)	55.4 (12.7)	58.8 (8.6)	51.4 (9.6)	59.2 (7.7)	58.4 (8.4)	75.6 (3.5)	76.4 (3.6)	56.3 (5.4)	55.0 (5.8)
Height (cm)	162.3 (7.3)	162.1 (7.3)	166.6 (7.6)	166.2 (9.1)	167.1 (9.4)	168.6 (9.6)	171.2 (8.9)	171.2 (9.4)	168.8 (9.2)	165.5 (9.2)	170.4 (7.8)	170.8 (8.4)	166.3 (9.0)	166.0 (9.4)	171.5 (7.0)	172.4 (6.9)
Weight (kg)	59.5 (10.6)	59.9 (11.1)	71.9 (11.1)	71.9 (13.3)	75.0 (14.9)	71.8 (14.0)	80.7 (15.5)	76.8 (15.2)	77.3 (13.9)	71.5 (13.4)	80.7 (13.3)	78.9 (13.4)	74.7 (13.1)	73.4 (13.0)	76.5 (11.3)	76.6 (10.6)
BMI (kg/m2)	22.5 (3.5)	22.8 (3.8)	25.9 (4.0)	26.1 (4.8)	26.8 (4.5)	25.2 (4.2)	27.2 (4.4)	26.1 (4.3)	27.1 (4.1)	26.1 (4.4)	27.8 (4.1)	27.0 (3.9)	27.0 (4.0)	26.6 (4.1)	26.0 (3.3)	25.7 (3.0)
WHR (-)	0.97 (0.07)	0.95 (0.07)	0.97 (0.06)	0.95 (0.06)	NR	NR	NR	NR	0.90 (0.09)	0.84 (0.09)	0.95 (0.08)	0.91 (0.09)	NR	NR	NR	NR
SBP (mmHg)	120.9 (23.0)	121.8 (18.3)	126.8 (20.2)	128.5 (17.1)	147.4 (22.6)	136.2 (21.9)	142.7 (21.9)	138.4 (20.4)	141.3 (20.2)	130.3 (18.9)	143.8 (20.8)	140.1 (19.8)	152.7 (23.0)	157.1 (21.4)	137.4 (17.6)	134.3 (17.7)
DBP (mmHg)	79.7 (13.8)	78.3 (10.3)	80.4 (11.2)	81.2 (9.8)	86.2 (12.2)	83.5 (12.2)	80.9 (12.7)	82.6 (11.2)	85.2 (10.9)	81.0 (10.7)	85.1 (11.1)	84.2 (11.0)	81.9 (12.0)	84.7 (11.6)	84.7 (10.2)	83.1 (10.4)
LDL (mmol/l)	3.18 (1.03)	2.70 (0.85)	3.22 (1.13)	2.77 (1.02)	4.08 (1.15)	3.65 (1.16)	2.89 (1.10)	3.31 (0.95)	4.77 (1.11)	4.16 (1.08)	3.93 (0.97)	3.76 (0.91)	3.72 (0.74)	3.78 (0.80)	4.99 (0.45)	4.60 (0.45)
HDL (mmol/l)	0.85 (0.21)	0.87 (0.22)	0.91 (0.27)	0.93 (0.29)	1.48 (0.49)	1.61 (0.50)	1.36 (0.45)	1.65 (0.51)	1.27 (0.38)	1.49 (0.41)	1.18 (0.34)	1.30 (0.35)	1.20 (0.32)	1.32 (0.37)	1.09 (0.24)	1.15 (0.24)
Triglycerides (mmol/l)	2.14 (1.34)	2.27 (1.32)	2.27 (1.40)	2.34 (1.40)	2.12 (1.30)	1.75 (1.13)	1.79 (1.22)	1.77 (1.19)	1.89 (1.18)	1.32 (0.88)	1.98 (1.25)	1.66 (1.08)	1.61 (0.76)	1.47 (0.65)	1.92 (0.78)	1.79 (0.75)
Total cholesterol (mmol/l)	5.13 (1.12)	4.71 (0.99)	5.06 (1.35)	4.66 (1.30)	6.23 (1.28)	6.05 (1.28)	4.86 (1.28)	5.73 (1.06)	6.41 (1.17)	5.91 (1.12)	6.10 (1.11)	5.92 (1.05)	5.57 (0.84)	5.68 (0.90)	7.03 (0.59)	7.00 (0.59)

Numbers are mean (SD), NR=Not recorded

eTable 4: Characteristics of participants from the Exome+ consortium by carriage of loss of function (LoF) variants

	European		South Asians	
	At least 1 LoF variant	No LoF variants	At least 1 LoF variant	No LoF variant
N	84	54,145	80	27,747
Male (n (%))	41 (48.8)	43 (51.2)	69 (86.3)	22,616 (81.5)
Age (years)	55.8 (11.9)	57.6 (11.9)	53.2 (10.1)	54.3 (10.0)
Height (cm)	168.0 (9.1)	168.8 (9.4)	165.7 (7.6)	165.5 (8.4)
Weight (kg)	74.8 (13.5)	75.7 (14.5)	66.4 (14.5)	69.4 (13.0)
BMI (kg/m^2)	26.6 (4.6)	26.5 (4.3)	24.1 (4.7)	25.3 (4.5)
WHR (-)	0.87 (0.10)	0.88 (0.10)	0.96 (0.06)	0.96 (0.06)
SBP (mmHg)	138.6 (23.0)	138.8 (21.1)	122.2 (18.7)	126.4 (19.3)
DBP (mmHg)	83.6 (12.5)	83.3 (11.4)	79.0 (10.0)	80.5 (10.9)
LDL (mmol/l)	3.88 (1.17)	3.81 (1.22)	2.84 (1.00)	2.97 (1.07)
HDL (mmol/l)	1.34 (0.40)	1.44 (0.46)	0.93 (0.30)	0.91 (0.27)
Triglycerides (mmol/l)	1.79 (1.18)	1.71 (1.13)	2.09 (1.53)	2.29 (1.38)
Total cholesterol (mmol/l)	5.78 (1.15)	5.86 (1.25)	4.79 (1.16)	4.86 (1.29)

Numbers are mean (SD)

eTable 5: Study-level characteristics of studies of rs76863441 (Val279Phe) and CHD or cardiovascular risk factors

Study/Author name	Country	Control population	Case definition	N cases	N controls	Overall minor allele frequency	Mean age	% Male	Lp-PLA2 activity assay	Genotyping method	Phenotypes reported by genotype*					
											Lp-PLA2 activity	Lp-PLA2 mass	Blood pressure	Lipids	Inflammation	Glucose /Diabetes
Studies with information on V279F and CVD																
Yamada 1998 & 2000$	Japan	Healthy hospital based*	MI or stroke	2517	6076	17%	56	66	Spec.	PCR	√		√	√		√
Hohda 2003	Japan	General population	MI	136	218	18%	47	76	NA	PCR						
Liu 2006	Taiwan	Healthy hospital based*	MI before age 45	200	200	17%	41	84	NA	Puregene DNA Isolation Kit						
Li 2011	China	Healthy hospital based*	AP (with stenosis > 50%) /MI	804	482	6%	61	69	NA	TaqMan						
Hou 2009 (Beijing atherosclerosis study)	China	General population	Non-fatal MI, CAD defined by stenosis (>70%)	810	914	5%	53	76	CAM	PCR	√					
Jang 2006 & 2011 (Study subset 1 & 2)	South Korea	Healthy volunteers	MI, CAD defined by stenosis (>50%)	5874	5222	12%	55	75	Spec.	TaqMan	√			√		
Takeuchi 2012	Japan		MI or angina with stenosis >75%	806	1335	20%**	66	64	NA	GWAS-Illumina						
Studies with information on V279F and CHD risk factors																
Ichihara 1998	Japan	Healthy hospital based	NA	NA	226	13%	54	76	Spec.	PCR	√					
Yoshida 1998	Japan	Healthy hospital based*	NA	NA	270	18%	61	56	Spec.	PCR	√					
Satoh 1999	Japan	General population	NA	NA	111	18%	-	-	Spec.	-	√					
Osoegawa 2004	Japan	General population	NA	NA	82	14%	-	-	Spec.	PCR	√					
Unno 2006	Japan	Healthy hospital based*	NA	NA	158	17%	71	86	TCA	PCR	√					
Zhang 2007	Japan	Healthy hospital based*	NA	NA	2018	17%	58	55	Azwell	PCR	√		√	√	√	
Wang 2009 (Shimane study)	Japan	Healthy hospital based*	NA	NA	800	19%	64	40	Cayman	PCR/Taqman	√		√	√	√	
Paik 2010	Korea	Healthy hospital based*	NA	NA	2914	13%	57	40	Radiometric	SNaPShot assay kit	√	√	√	√	√	√
Fan 2010	China	Hospital Infertile otherwise healthy	NA	NA	148	4%	28	0	TCA	PCR	√		√	√		

*Attending routine check up or screening test and found to lack any serious disorders; **Figures for Takeuchi relate to rs1805018 which is a proxy of rs16874954 in Japanese (r^2=0.94) + stenosis refers to stenosis >50% in at least one major coronary artery, except in the Beijing atherosclerosis study where it is at least 70%. $Non-overlapping subjects obtained via a data request. AP=angina pectoris; CAD=coronary artery disease; CAM=calorimetric assay method; MI=myocardial infarction; PAD=Peripheral arterial disease; PCR=polymerase chain reaction ; Spec.=Spectophometric; TCA=tricalorimetric assay

eTable 6: Number of cases and controls carrying loss of function variants

Study	Participants with loss of function variants		Participants without loss of function variants	
	Cases	Controls	Cases	Controls
Gln287Ter				
CCHS	1	4	2,019	6,083
CIHDS/CGPS	7	8	8,072	10,359
EPIC-CVD	9	10	9,801	10,960
MORGAM	0	1	2,153	2,117
PROSPER	0	0	641	638
WOSCOPS	0	1	659	686
MICAD consortium	32	51	36,155	64,581
**TOTAL**	**49**	**75**	**59,500**	**95,424**
rs142974898				
CCHS	0	0	2,020	6,087
CIHDS/CGPS	0	0	8,079	10,367
EPIC-CVD	3	4	9,807	10,966
MORGAM	1	0	2,152	2,118
PROSPER	0	0	641	638
WOSCOPS	0	0	659	687
MICAD consortium	Not assayed			
European total	4	4	23,358	30,863
BRAVE	0	0	2,971	2,782
PROMIS	0	0	10,135	11,928
South Asian total	0	0	13,106	14,710
**TOTAL**	**4**	**4**	**36,464**	**45,573**
Arg82His				
CCHS	0	2	2,020	6,085
CIHDS/CGPS	1	0	8,078	10,367
EPIC-CVD	2	8	9,808	10,962
MORGAM	2	2	2,151	2,116
PROSPER	1	0	640	638
WOSCOPS	2	0	657	687
MICAD consortium	14	23	36,173	64,609
European total	22	35	59,527	95,464
BRAVE	13	6	2,958	2,776
PROMIS	10	14	10,125	11,914
South Asian total	23	20	13,083	14,690
**TOTAL**	**45**	**55**	**72,610**	**110,154**
Val279Phe				
CCHS	0	1	2,020	6,086
CIHDS/CGPS	0	2	8,079	10,365
EPIC-CVD	5	6	9,805	10,964
MORGAM	0	1	2,153	2,117
PROSPER	0	0	641	638
WOSCOPS	0	0	659	687
MICAD consortium	11	20	36,176	64,612
European total	16	30	59,533	95,469

BRAVE	10	10	2,961	2,772
PROMIS	7	10	10,128	11,918
South Asian total	17	20	13,089	14,690
**TOTAL**	**33**	**50**	**72,622**	**110,159**
